# Vasculitic emergencies in the intensive care unit: a special focus on cryoglobulinemic vasculitis

**DOI:** 10.1186/2110-5820-2-31

**Published:** 2012-07-19

**Authors:** Mohamad Zaidan, Eric Mariotte, Lionel Galicier, Bertrand Arnulf, Véronique Meignin, Jérôme Vérine, Alfred Mahr, Élie Azoulay

**Affiliations:** 1 Department of Medical Intensive Care Unit, Hôpital Saint-Louis, AP-HP, Université Paris-7 Diderot, Paris, France; 2 Department of Clinical Immunology, Hôpital Saint-Louis, AP-HP, Université Paris-7 Diderot, Paris, France; 3 Department of Immuno-Hematology, Hôpital Saint-Louis, AP-HP, Université Paris-7 Diderot, Paris, France; 4 Department of Pathology, Hôpital Saint-Louis, AP-HP, Université Paris-7 Diderot, Paris, France; 5 Department of Internal Medicine, Hôpital Saint-Louis, AP-HP, Université Paris-7 Diderot, Paris, France; 6 AP-HP, Hôpital Saint-Louis, Medical ICU, University Paris-7 Paris-Diderot, UFR de Médecine, 1 avenue Claude Vellefaux, 75010 Paris, France

**Keywords:** Cryoglobulinemia, Cryoglobulinemic vasculitis, Acute respiratory failure, Acute kidney injury, Vasculitis, Systemic disease

## Abstract

Vasculitis is characterized by the infiltration of vessel walls by inflammatory leukocytes with reactive damage and subsequent loss of vessel integrity. The clinical course of systemic vasculitis may be punctuated by acute life-threatening manifestations that require intensive care unit (ICU) admission. Furthermore, the diagnosis may be established in the ICU after admission for a severe inaugural symptom, mostly acute respiratory failure. Among the systemic vasculitides, cryoglobulinemic vasculitis (CV) has been rarely studied in an ICU setting. Severe CV-related complications may involve the kidneys, lungs, heart, gut, and/or central nervous system. The diagnosis of CV in the ICU may be delayed or completely unrecognized. A high level of suspicion is critical to obtain a timely and accurate diagnosis and to initiate appropriate treatment. We describe severe acute manifestations of CV based on six selected patients admitted to our ICU. That all six patients survived suggests the benefit of prompt ICU admission of patients with severe CV.

## Review

### Introduction

Vasculitis is defined by the presence of inflammatory leukocytes in vessel walls with reactive damage and subsequent loss of vessel integrity, which can lead to bleeding, tissue ischemia, and necrosis [1,2]. The natural course of as-yet-undiagnosed and untreated systemic vasculitis may result in acute and life-threatening manifestations that require management in an intensive care unit (ICU) [3-8].

Cryoglobulinemic vasculitis (CV) is a small-vessel systemic vasculitis that results from the deposition of cryoglobulins on the vessel walls, activating the complement cascade [9-15]. Cryoglobulinemia is defined by the presence in the serum of one or more immunoglobulins (Igs) that undergo reversible precipitation at temperatures below 37°C [9-11,15]. The 1974 Brouet classification distinguishes three cryoglobulin types based on the immunoglobulin composition of the cryoprecipitate (Additional file 1: Table S1) [14]**.**

Clinically, the classical triad of purpura, weakness, and arthralgia is present in up to 80–90% of patients and may be associated with a broad spectrum of abnormalities, including other skin and joint manifestations, as well as involvement of the kidneys, digestive tract, peripheral nervous system, and salivary glands (Additional file 1: Table S2) [9-19]. Of note, serum cryoglobulins may be missed despite repeated testing and a typical cryoglobulinemic syndrome. Immune-complex-mediated, necrotizing, or leukocytoclastic small vessel-vasculitis is the pathological hallmark [9-11,15].

**Figure 1:**
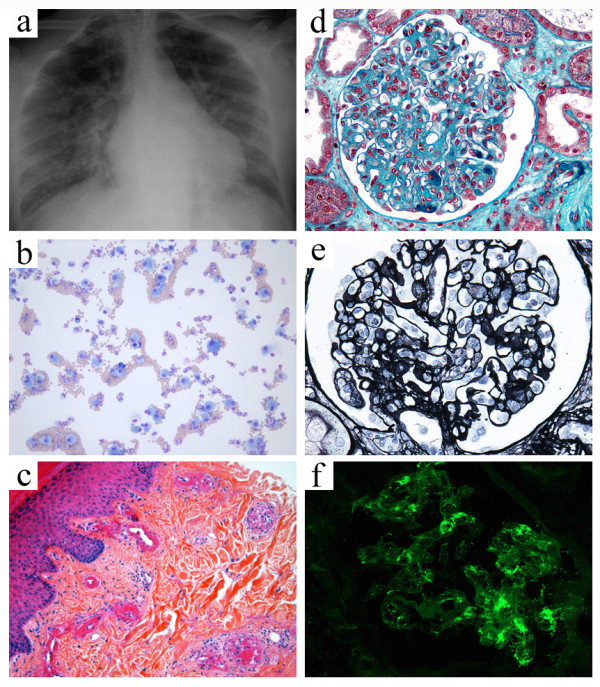
**Widespread fulminant cryoglobulinemic vasculitis is defined as involvement of the skin and at least two other organs, such as illustrated by the following case.** A 50-year-old woman presented with fever, bilateral purpuric lesions, dyspnea, and acute kidney injury. The bronchoalveolar lavage showed severe hemorrhage with predominance of macrophages and neutrophils (**a**, May-Grünwald stain, x200). The skin biopsy revealed typical features of leukocytoclastic vasculitis with intraluminal thrombosis and fibrinoid-type necrosis associated with infiltration by neutrophils of the vessel walls in the dermis (**b**, hematoxylin-eosin-saffron stain, x200). The kidney biopsy displayed membranoproliferative glomerulonephritis. The glomeruli showed mesangial expansion by increased mesangial cell number and matrix, and some intracapillary "protein thrombi" (**c**, Masson’s trichrome stain, x400). A mild endocapillary hypercellularity and duplication of the glomerular basement membrane with “double contours” were also observed (**d**, Jones stain, x400). Immunofluorescence study showed granular IgG-positive deposits with *lambda* light-chain isotype restriction localized to the glomerular capillary wall, mesangium, and intracapillary thrombi (**e,** IgG Immunofluorescence staining, x400, *lambda* staining not shown). IgM, IgA, and *kappa* staining were all negative (data not shown). Lambda staining was comparable to IgG staining (data not shown), which was consistent with renal involvement of type I cryoglobulinemia (IgG *lambda*). Finally, the diagnosis was widespread cryoglobulinemic vasculitis with skin, lung, and kidney involvement secondary to monoclonal gammopathy of undetermined significance associated with Sjögren’s syndrome.

The clinical course of CV is uneventful in more than 50% of patients [9-11,15]. Fulminant, life-threatening flares are rare but must be diagnosed and treated promptly [20-23]. Widespread vasculitis, which is defined as involvement of the skin and at least two other organs, may develop (Table 1; Figure 1) [9-11,15,20-23]. In a retrospective study of 209 consecutive patients with CV, 29 patients had potentially life-threatening CV-related manifestations, of which 59% were present at onset [20]. These manifestations were associated with a higher mortality rate and included renal failure due to cryoglobulinemic glomerulonephritis, intestinal vasculitis, pulmonary hemorrhage, and central nervous system involvement [20]. The mortality rate reached 100% for patients with pulmonary hemorrhage or intestinal ischemia. The diagnosis of CV in the ICU may be delayed or be completely unrecognized. A high level of suspicion is critical to obtain a timely and accurate diagnosis and to initiate appropriate treatment. The scope of this review is to illustrate the potentially life-threatening nature of CV through the description of six selected patients admitted to our ICU. Written informed consent was obtained from the patient for publication of this report and any accompanying images. The clinical vignette of five patients is detailed, and one patient is only depicted in Figure 1. That all six patients survived argues strongly in favor of prompt ICU admission of patients with severe CV.

**Table 1:** **Acute life-threatening manifestations of cryoglobulinemic vasculitis **[[9-14,20-23]]

	
**Widespread vasculitis**	- Multiple organ involvement, including the skin and at least two other organs (kidney, gut, lung, CNS)
**Renal involvement**	- AKI and/or oliguria
	- Glomerulonephritis :
	- Membranoproliferative glomerulonephritis
	- Mesangial glomerulonephritis
	- Focal and segmental glomerulonephritis
**Lung involvement**	- Subclinical alveolitis
	- Interstitial lung fibrosis
	- Diffuse alveolar hemorrhage
**Heart involvement**	- Acute heart failure
	- Acute coronary syndrome
**Digestive tract involvement**	- Ischemic bowel vasculitis
	- Gastrointestinal hemorrhage
	- Acute intraabdominal organ injury (pancreatitis, cholecystitis)
**CNS involvement**	- Stroke
	- Encephalopathy with impaired cognitive function
	- Brain hemorrhage
	- Spinal cord involvement
**Other**	- Hyperviscosity syndrome
	- caused by high levels of cryoglobulins
	- combines acute respiratory distress, visual disturbances and retinal hemorrhage, encephalopathy with impaired cognitive function, and AKI
	- B-cell lymphoproliferative disorders and related complications
	- Sepsis, bacterial infections, and related complications
	- Liver failure in HCV-related mixed cryoglobulinemia (acute-on-chronic hepatitis and cirrhosis)

HCV = hepatitis C virus; AKI = acute kidney injury; CNS = central nervous system.

### Illustrative review

#### Two patients admitted to the ICU for acute respiratory failure due to CV-related renal involvement

##### Case 1 renal involvement

**Figure 2:**
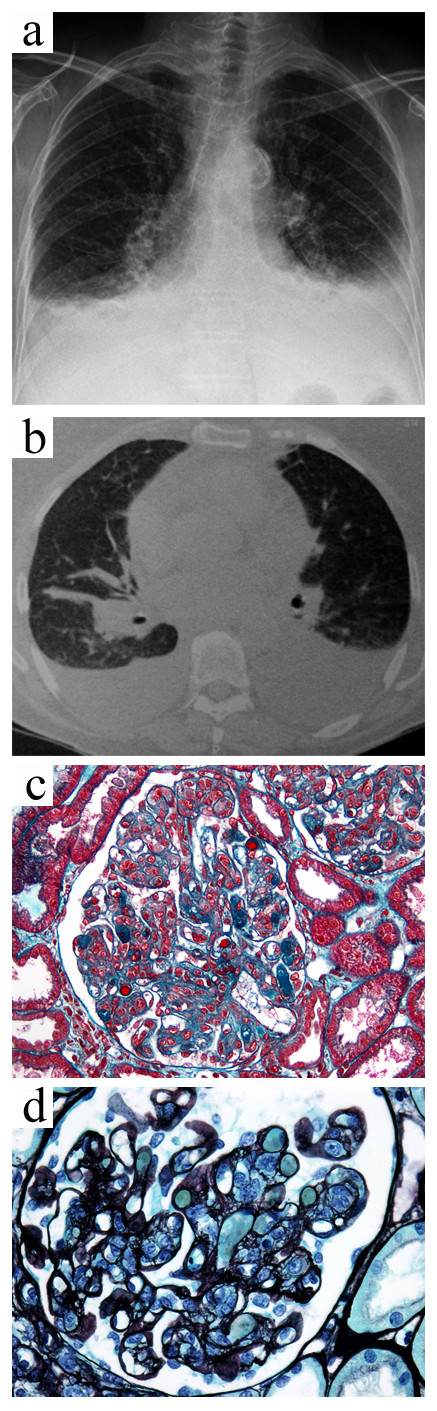
**Case 1.** Primary Sjögren’s syndrome-related mixed cryoglobulinemia with peripheral neuropathy, skin lesions, and glomerulonephritis. The chest radiograph (**a**) and computed tomography scan (**b**) showed bilateral pleural effusion and alveolar infiltrates. The kidney biopsy showed membranoproliferative glomerulonephritis with abundant infiltrating monocytes and intracapillary "protein thrombi" (**c**, Masson’s trichrome stain, x400) and “double contours” (**d**, Jones’ silver stain, x400).

An 82-year-old female with an unremarkable medical history presented with recent onset of purpura, edema, and paresthesia with burning sensations in her extremities. Her condition worsened rapidly with acute respiratory distress and oliguria. At admission to the ICU, her blood pressure was 195/67 mmHg and she had bilateral crackles by lung auscultation. Chest computed tomography (CT) showed a bilateral pleural effusion and alveolointerstitial infiltrates (Figures 2a-b). Echocardiography indicated left ventricular diastolic dysfunction. The cryoglobulin test was positive for type II mixed cryoglobulinemia with a monoclonal IgM *kappa*. The serum C3 level was normal, but the serum C4 level was low. Serological tests for hepatitis C and B and human immunodeficiency virus were negative. A skin biopsy revealed leukocytoclastic vasculitis, and a kidney biopsy membranoproliferative glomerulonephritis (Figures 2c-d). The patient complained from xerostomia, and antinuclear and anti-SSA antibodies were positive. A minor salivary gland biopsy displayed a mononuclear cell infiltrate consistent with Sjögren’s syndrome. The diagnosis was primary Sjögren’s syndrome-related mixed cryoglobulinemia with peripheral neuropathy, skin lesions, and glomerulonephritis. The patient received oral prednisone (1 mg/kg/day) and four weekly infusions of rituximab (375 mg/kg/m² per week). She improved and her renal function recovered *ad integrum*.

##### Case 2 renal involvement

**Figure 3:**
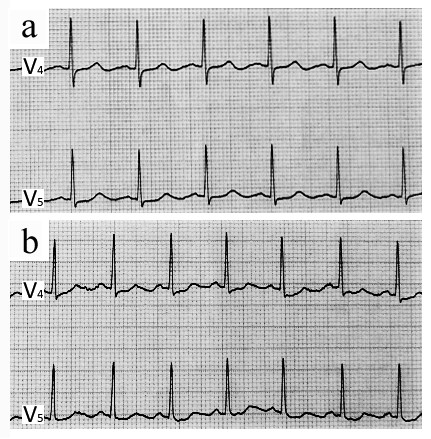
**Case 2.** The electrocardiogram was normal at admission (**a**) and showed minor nonspecific changes (ST segment depression) during an episode of chest pain at rest (**b**).

A 66-year-old female, with a history of type I CV due to a low-grade B-cell lymphoma 2 years before, was admitted to the ICU for acute respiratory failure. Her temperature was 36°C, heart rate 132 beats per minute, blood pressure 220/100 mmHg, and oxygen saturation 80%. The physical examination showed peripheral edema, purpura over the lower limbs, and bilateral lung crackles. She was rapidly intubated. Laboratory findings were as follows: hemoglobin, 8.8 g/dL; leukocyte count, 16,800/mm^3^; platelet count, 292,000/mm^3^; serum creatinine, 615 μmol/L; serum urea, 24 mmol/L; proteinuria in the nephrotic range; and hematuria. She experienced two episodes of nitroglycerin-responsive chest pain with slight troponin elevation and ST segment depression (Figure 3). The test for cryoglobulin was positive for type I cryoglobulinemia (IgG *kappa*), and the C4 level was low with undetectable total complement activity. These findings were consistent with a CV flare associated with low-grade B-cell lymphoma responsible for renal, skin lesions, and unstable angina. Steroids were initiated with daily plasma exchanges in addition to symptomatic treatment and hemodialysis. The patient was given low-dose aspirin and clopidogrel, combined with rituximab (375 mg/m²/week), and cyclophosphamide pulses (600 mg/m²/month). The cryoglobulin fell to undetectable levels. Her condition improved, but the renal function did not recover.

*Renal involvement* is the most common, severe, CV-related visceral manifestation, seen in more than one-third of cases [9-18]. Of 29 patients with life-threatening CV, 55% of the life-threatening episodes were due to CV-related glomerulonephritis [20]. The renal symptoms typically consisted of mild proteinuria, microscopic hematuria, and hypertension [24-27]. More than 50% of patients had mild renal failure [24-27]. Nephrotic syndrome, nephritic syndrome, and acute kidney injury (AKI) have been reported in 20%, 14–25%, and 9% of patients, respectively [24,26]. Fluid overload, oliguria, and metabolic disorders may require urgent renal replacement therapy, as illustrated by our second case [20,23]. The main renal biopsy finding is type 1 membranoproliferative glomerulonephritis (70–80% of patients) [24-27]. Mesangial proliferative glomerulonephritis and focal and segmental glomerulonephritis also have been described [13,24-26]. Renal failure may also result from nonglomerular lesions due to heart failure, sepsis, or hemorrhagic shock. The main short-term complications are AKI, oliguria, fluid overload with acute respiratory distress, and metabolic disorders. End-stage renal disease requiring long-term dialysis occurs in less than 15–30% of patients, and only after a course of 10 years in half of the cases [24,25]. In a retrospective study of 105 patients with CV-related nephropathy and a mean disease duration of 11 years, factors predicting end-stage renal failure were age >50 years, splenomegaly, cryocrit >10%, low C3 level, recurrent purpura, initial serum creatinine >136 μmol/L, and HIV coinfection [24]. Male gender may be correlated with a higher risk for requiring dialysis [26]. In a series of 146 patients with cryoglobulinemic glomerulonephritis, age, serum creatinine, and proteinuria at the time of kidney biopsy correlated independently with end-stage renal failure [26]. CV nephropathy may affect survival severely [9-11,15,19,24-26]. Retrospective studies have pointed to renal involvement as a marker of poor prognosis [9-11,15,19,24-26]. Interestingly, Terrier et al. have shown recently that a glomerular filtration rate <60 ml/min/1.73 m² at presentation, but not hematuria and proteinuria >1 g/day, was significantly associated with a poor prognosis in multivariate analysis [28]. Of 105 patients with essential mixed cryoglobulinemia and renal involvement, 49% were alive 10 years after the renal biopsy—the main cause of death was cardiovascular disease—whereas 10-year survival after CV nephropathy onset was approximately 80% in a more recent study [24,26]. Cardiovascular disease, infections, liver failure, non-Hodgkin’s lymphoma, and other malignancies were the main causes of death [19,24-26].

#### Two patients admitted to the ICU for acute respiratory failure due to CV-related pulmonary or cardiac disease

##### Case 3 pulmonary disease

**Figure 4:**
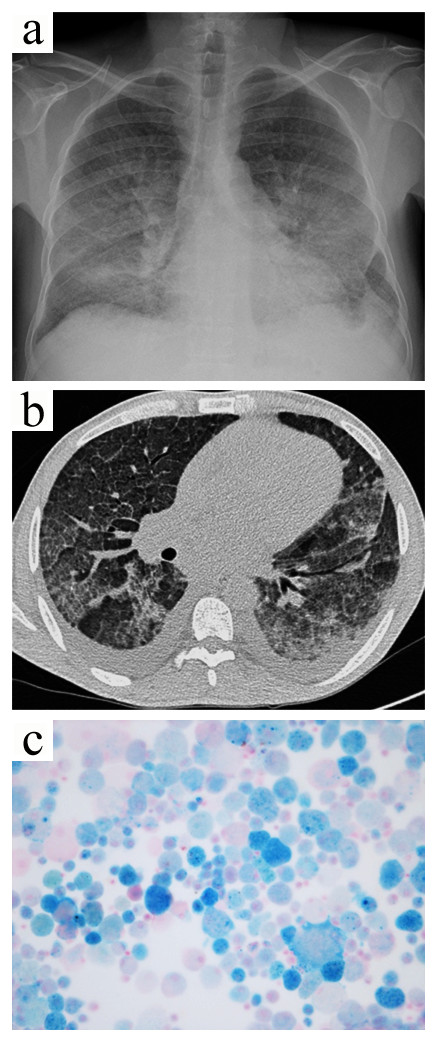
**Case 3.** The chest radiograph (**a**) showed diffuse nonspecific alveolar infiltrates. Chest computed tomography (**b**) revealed areas of ground-glass attenuation interspersed with normal areas, as well as a bilateral pleural effusion. The bronchioloalveolar lavage showed many blue hemosiderin-laden macrophages consistent with diffuse alveolar hemorrhage (**c**, Perls’ stain, x400).

A 47-year-old male was referred to our hospital for exertional dyspnea and diffuse vascular purpura. At admission, his temperature was 36.7°C, his heart rate was 64 beats per minute, his blood pressure was 174/78 mmHg, and his oxygen satu-ration was 89%. Laboratory findings were as follows: hemoglobin, 7.8 g/dL; leukocyte count 3,600/mm^3^; platelet count, 179,000/mm^3^; serum creatinine, 278 μmol/L; serum urea, 24.6 mmol/L; and serum albumin, 30 g/L. The ratio of urinary protein to creatinine in random samples was 1,700 mg/mmol. Serological testing for HCV was positive. The serum C3 level was normal and the C4 level was 8 mg/dl (normal, 16–40). Rheumatoid factor was positive and serum type II cryoglobulinemia was found. The renal biopsy showed membranoproliferative glomerulonephritis. The patient received two weekly infusions of rituximab (375 mg/m²/week). Acute respiratory distress suddenly developed. At admission to the ICU, lung auscultation found bilateral crackles. The peripheral edema and skin lesions had worsened. Chest radiography and CT showed bilateral ground-glass opacities and alveolar infiltrates (Figure 4a-b). The bronchoalveolar lavage fluid contained 59·10^4^ cells/mL with 84% macrophages of which 90% were hemosiderin-laden alveolar macrophages (Figure 4c). Cultures were negative. The diagnosis was CV-related pulmonary-renal syndrome with diffuse alveolar hemorrhage and membranoproliferative glomerulonephritis. He received three intravenous steroid pulses (1,000 mg/day) followed by oral prednisone (50 mg/day) in addition to intravenous cyclophosphamide pulses (600 mg/m²/month). A prompt improvement was noted with resolution of the purpura and partial recovery of renal function. Pegylated interferon alfa-2a and ribavirin therapy decreased the HCV load to undetectable levels. The cyclophosphamide was discontinued and the prednisone tapered.

*Lung involvement* in CV is mainly characterized by subclinical alveolitis, which occasionally leads to interstitial lung fibrosis [9,29]. Although only scarce data are available in the literature, patent pulmonary involvement has been associated with poor survival in patients with CV [28]. In the series by Ramos-Casals et al., the mortality rate reached 100% for patients with pulmonary hemorrhage [20]. Acute severe pulmonary manifestations in patients with CV include (1) pulmonary edema, due to fluid overload caused by kidney and heart failure, (2) bronchopulmonary and systemic infections, (3) diffuse alveolar hemorrhage, and (4) hyperviscosity-related pulmonary lesions. Infections, although not specific of CV, may be precipitated by immunodeficiency related to the underlying disorder (most notably hematological malignancies) and drugs used to treat CV (steroids and immunosuppressive agents) [9-11,15]. Retrospective studies indicate that infections are among the leading causes of death in patients with CV, after cardiovascular events [19,24-26]. Acute respiratory distress is usually related to renal and/or heart failure with fluid overload and pulmonary edema, rather than to specific lung lesions, namely, diffuse alveolar hemorrhage and hyperviscosity-related pulmonary lesions [9,12,13,15,20,23,30-33]. Diffuse alveolar hemorrhage was the most common reason for ICU admission of patients with small-vessel vasculitis in the retrospective series of Khan et al., although none of the 28 patients displayed CV [5]. Patients with diffuse alveolar hemorrhage generally present with acute hypoxemic respiratory failure or hemoptysis and anemia [5,30-34]. Chest radiography and CT show diffuse pulmonary infiltrates, suggesting ongoing alveolitis [5,30-34]. When performed, bronchoscopy usually reveals blood in the bronchoalveolar lavage fluid. The pathological examination may reveal intraalveolar red blood cells, fibrin, and eventually hemosiderin-laden macrophages, which may take up to 48 or 72 h to accumulate [30,34]. Diffuse alveolar hemorrhage is associated with a wide spectrum of disorders, including systemic vasculitis and connective tissue disease [34]. Diffuse alveolar hemorrhage occurs in less than 5% of patients with mixed cryoglobulinemia, irrespective of HCV status, and is generally associated with CV-related glomerulonephritis [30-33]. Therefore, CV should be on the list of differential diagnoses of pulmonary-renal syndrome [30,34]. Our case contrasts with other reported cases, because diffuse alveolar hemorrhage is usually associated with a poor outcome [30]. It is worth noting that the lung manifestations occurred shortly after the administration of rituximab, a drug whose reported adverse events include interstitial pneumonitis [35-37]. Interestingly, in patients with HCV-related CV, rituximab may form a complex with IgM kappa mixed cryoglobulin, thereby inducing an acute systemic flare of CV [38].

##### Case 4 cardiac disease

A 40-year-old female with chronic HCV infection was admitted to the ICU for acute bronchospastic respiratory distress. The temperature was 39.5°C, the heart rate was 170 beats per minute, the blood pressure was 200/120 mmHg, and the respiratory rate was 45 breaths per minute. The troponin level was slightly increased (0.13 μg/L, N < 0.02 μg/L), and the electrocardiogram showed T-wave inversion with normal ST segments. Endotracheal ventilation was started. Community-acquired pneumonia was suspected and empirical broad-spectrum antibiotic therapy was initiated. The patient also received bronchodilators and oral prednisone (1 mg/kg/day for 5 days). She improved and was extubated. However, she experienced an episode of abdominal pain and acute anemia (7.5 g/dL). Upper gastrointestinal endoscopy showed a cardial ulcer with no active bleeding. Skin ulcers developed over the legs, as well as nephrotic syndrome with peripheral edema and microscopic hematuria but no renal failure.

The diagnosis was HCV-related type II cryoglobulinemia involving the skin, joints, and kidneys, and probably the gut. Steroid therapy was resumed. Acute respiratory distress developed rapidly. The electrocardiogram showed T-wave inversion with normal ST segments. Endotracheal ventilation was initiated. A skin rash with purpura was noted after the administration of cold serum to measure cardiac output during Swan-Ganz catheter insertion. Echocardiography showed left ventricular diastolic dysfunction. The bronchoalveolar lavage fluid contained 26·10^4^ cells/mL with 3.5% neutrophils and no hemosiderin-laden alveolar macrophages. Cultures were negative. The creatinine serum level increased to 127 μmol/l. Urinalysis showed hematuria and proteinuria in the nephrotic range. The diagnosis was respiratory failure due to CV-related heart failure precipitated by hypertensive crisis and renal involvement. Plasma exchanges were initiated in addition to symptomatic treatment. Replacement liquids were warmed before administration. Four weekly rituximab infusions (375 mg/m²/week) were administered. Ribavirin was added to pegylated interferon alfa-2a to control chronic HCV infection. The skin lesions resolved and the renal function recovered. Coronary angiography performed a few weeks later showed normal coronary arteries, suggesting involvement of the coronary microcirculation, which was consistent with CV-related heart involvement.

*CV-related cardiac disease* is a rare but life-threatening manifestation of CV [9,16,23,39-41]. Systemic disease with respiratory distress and renal involvement is usually present in patients with CV-related cardiac disease. Acute cardiac involvement should be distinguished from long-term cardiovascular complications, which are among the leading causes of death in patients with CV [9,16,19,23,24,26]. Moreover, the previous cardiovascular status should be considered [40]. CV-related cardiac disease usually occurs concomitantly with other severe organ manifestations [39,41]. Heart dysfunction with pulmonary edema is the main presentation. As reported in our patients, echocardiography may reveal diastolic and systolic dysfunction with low cardiac output and segmental dyskinesia or akinesia. Precipitating factors include: AKI; hypertensive crisis, which is usually severe and controllable only by using multiple antihypertensive medications; and coronary heart disease [9,23]. Coronary heart disease is difficult to assess in patients with CV, because typical chest pain is not always present. The manifestations may consist only in mild troponin elevation or subtle changes in T waves or ST segments. CV-related coronary heart disease usually involves the coronary microcirculation, sparing the major arteries, which are usually normal by angiography [39]. The etiologic and symptomatic treatment includes antiplatelet agents, anticoagulation, and beta blockers, in addition to steroids and plasma exchanges. Cardiac biomarkers and the electrocardiogram should be monitored closely until the symptoms resolve and the laboratory data return to normal. After treatment, the imaging studies, particularly the echocardiogram, should be repeated to assess heart function recovery, as illustrated by our case 4 [41].

#### Patient admitted to the ICU with severe gastrointestinal involvement

##### Case 5 gastrointestinal involvement

**Figure 5:**
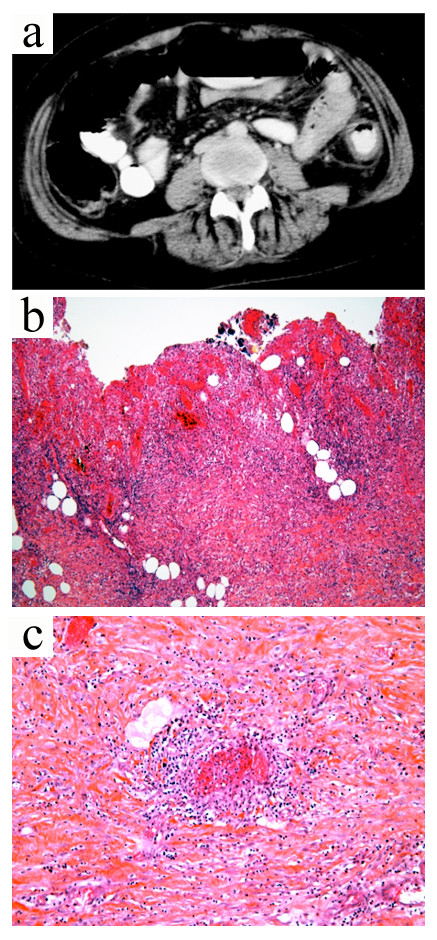
**Case 4.** Contrast-enhanced computed tomography of the abdomen (**a**) showed circumferential wall thickening and heterogeneous enhancement with layers of low attenuation consistent with colonic edema. Pathological analysis of colectomy specimen revealed severe necrosis with complete loss of crypts, hemorrhage and infiltration of the colon by inflammatory cells (**b**, hematoxylin-eosin-saffron stain, x200). Small-vessel vasculitis was observed with fibrinoid necrosis and vessel wall infiltration by neutrophils (**c**, hematoxylin and eosin stain, x400).

A 43-year-old female with a history of Sjögren’s syndrome-related type II cryoglobulinemia was hospitalized for recent onset of ulcers on her legs, acute abdominal pain, and peripheral edema. Urinalysis showed nephrotic-range proteinuria and hematuria, and the renal biopsy disclosed membranoproliferative glomerulonephritis. By abdominal CT, she had mild thickening of the left colonic wall consistent with ischemic colitis. Empirical antibiotics and three daily intravenous methylprednisolone pulses (1,000 mg/day) followed by prednisone (1 mg/kg/day) were administered. Her condition improved, and the steroids were tapered. Two weeks later, she experienced recurrent severe abdominal pain with high-grade fever. She was rapidly transferred to the ICU. Early colonoscopy showed numerous lesions of different ages, some of which were deep ulcerations without perforation, consistent with ischemic colitis. Empirical broad-spectrum antibiotics were initiated. Repeated physical examination demonstrated mild tenderness and peritoneal irritation over the left side of the lower abdomen. Emergency laparotomy revealed stercoral perforation of the colon. Colectomy followed by colostomy was performed. The pathological findings confirmed the diagnosis of CV-related ischemic colitis (Figure 5). In the meantime, mild dyspnea and a bilateral pleural effusion developed. The electrocardiogram and troponin levels were unchanged. Echocardiography indicated severe left ventricular dysfunction with septal akinesia. The diagnosis was a CV flare involving the skin, gut, kidneys, and heart. Intravenous cyclophosphamide pulse therapy (500 mg/m²) was added to the steroids and symptomatic treatment. A prompt improvement was noted, with resolution of the dyspnea and pleural effusion. She was discharged. Secondary bowel anastomosis was performed a few months later. Her cardiac abnormalities improved. Monthly intravenous cyclophosphamide pulses (500 mg/m²/month) were given for 9 months. The steroids were tapered to 5 mg/day and hydroxychloroquine was added to the long-term treatment regimen.

*Gastrointestinal involvement* is rare in patients with CV (5%) [9,13,15,16,19-21,42-44]. CV may cause ischemic bowel disease, gastrointestinal hemorrhage, peritonitis, and acute lesions of any of the intraabdominal organs, including the pancreas and gallbladder [13,20,21,42-44]. In a case-control study of 163 patients who had HCV-related CV and polyarteritis nodosa-like vasculitis, gastrointestinal involvement was observed in only four patients with histologicallydocumented CV [44]. In patients with HCV-related systemic vasculitis, gastrointestinal lesions may be related to CV or polyarteritis nodosa-like vasculitis, although the test for serum cryoglobulin is usually positive [44]. Pathological studies may assist in the differential diagnosis [44]. Severe manifestations include acute surgical complications (peritonitis/perforation, mesenteric infarction, and cholecystitis), intestinal bleeding, and pancreatitis [20,44]. Gastrointestinal lesions are associated with an increased likelihood of renal and cardiac involvement [44]. Early diagnosis and management can lead to a favorable outcome, as in our case [44].

#### Other potentially life-threatening manifestations of cryoglobulinemic vasculitis

*Neurological manifestations* consist chiefly of peripheral neuropathy, particularly sensory and mixed polyneuritis [9-15,45]. CV-related central nervous system manifestations, including stroke, brain hemorrhage, and encephalopathy, with impaired cognitive function are exceedingly rare [13,16,20,45-50]. Spinal cord involvement has been described [13]. The underlying mechanisms remain unclear but may involve either multiple, small, brain infarcts or small-vessel vasculitis [45].

*Hyperviscosity syndrome* due to high cryoglobulin levels is another rare but life-threatening complication of CV and is seen mainly in patients with lymphoproliferative disorders, chiefly Waldenström’s macroglobulinemia [9,10,12,51]. The clinical presentation may combine acute respiratory distress, visual disturbances and retinal hemorrhage, encephalopathy with impaired cognitive function, and AKI [10,51]. Hydration, steroids, and other immunosuppressive agents, as well as plasma exchange, should be initiated rapidly in patients with hyperviscosity syndrome [9-11,15,52,53].

*Liver failure* is a major concern in patients with HCV-related CV [9-13,15,19,20,23,24]. Acute-on-chronic liver failure or cirrhosis is the result of chronic active HCV infection rather than specific CV-related hepatic lesions [9,10,27].

*Severe infections* with septic shock may occur during the course of CV, particularly in patients receiving steroids and immunosuppressive agents or undergoing renal replacement therapy or plasma exchanges [19,24,26].

#### Management of severe acute CV-related manifestations

In clinical practice, particularly in the ICU, the management of life-threatening vasculitis is challenging [3,4,9]. A timely and accurate diagnosis is mandatory for the appropriate treatment to be implemented. The treatment has three targets: the symptoms, the cause, and the pathogenic mechanism [3,4,9-11,15,20,23,52]. In critically ill patients, the first-line treatment focuses on organ support and prevention of long-term organ dysfunction.

Treatments targeting the pathogenic mechanisms consist of steroids, immunosuppressive agents, and sometimes plasma exchange [4]. These treatments should be tailored to disease severity, to the nature and extent of organ dysfunction in each individual patient [4,9-11,15,52,54]. Although no data from controlled, randomized, clinical trials are available, guidelines have been developed [52]. In critically ill patients with CV, the first-line treatment aims at reversing the pathogenic mechanisms and consists of high-dose steroids (intravenous methylprednisolone pulses of 500–1000 mg/day for 3 days, followed by oral prednisone 1 mg/kg/day) and some of the time plasma exchanges (3 liters of plasma per exchange, three times a week for 2–3 weeks) [9-11,15,52,53]. Immunosuppressive drugs—cyclophosphamide (2 mg/kg/day orally or 600 mg/m²/month intravenously for 2–4 months) or rituximab (375 mg/m²/week for 4 weeks)—should be considered promptly [9-11,15,52,53]. Early plasma exchanges are mandatory in patients with hyperviscosity syndrome [9-11,15,52,53]. Plasma exchanges aim at reducing the levels of circulating immune complexes, especially those containing cryoglobulins, whereas steroids and immunosuppressive agents help to reduce the production of cryoglobulins and to limit the inflammatory process in the blood vessel wall [9-11,15,52,53]. Paradoxical precipitation of cryoglobulins has been reported after infusion of cold plasma, suggesting that replacement fluids for plasma exchange in CV should be warmed before infusion [55].

Identification of the underlying cause is mandatory and an etiology-based treatment is the mainstay of the long-term management [9-11,15,52,53]. Interestingly, in the study by Ramos-Casals et al. that reported a high mortality rate in patients with life-threatening CV, most patients were HCV-positive and received immunosuppressant agents without antiviral therapy, which could have been deleterious [20]. In case of HCV-related CV, the eradication of HCV infection is crucial [9-11,15,52,53]. Antiviral therapy for 12 months consisting of standard or pegylated interferon-alpha combined with ribavirin is the reference standard and should be given whenever possible [9-11,15,52,53]. Of note, ribavirin is best avoided if the glomerular filtration rate is <50 mL/min/1.73 m² to avoid hemolytic anemia [52]. Moreover, it has been reported that interferon initiation during acute CV onset may result in paradoxical exacerbation of CV-related manifestations [56]. Patients should be monitored closely for immune-mediated side effects of interferon therapy or ribavirin [52].

In a recent large study, Terrier et al. has showed that rituximab plus steroids had the greater therapeutic efficacy compared with steroids alone and alkalinating agents plus steroids to achieve complete clinical, renal, and immunological remission and a prednisone dosage <10 mg/day at 6 months [57]. However, this regimen was associated with severe infections, particularly when high doses of corticosteroids were used, whereas death rates did not differ between the therapeutic regimens [57]. Of note, only patients with underlying low-grade B-cell lymphoma or with refractory disease after rituximab and cyclosphamide as single agents should be administered combined rituximab plus cyclophosphamide therapy. Finally, patients with glomerulonephritis should receive angiotensin-converting enzyme inhibitors or angiotensin II receptor blockers and antihypertensive drugs to achieve the blood pressure and proteinuria targets established for patients with chronic kidney disease. This approach is crucial to minimize the long-term cardiovascular complications, which are the leading cause of death in patients with CV-related nephropathy [26,52].

## Conclusions

The natural course of systemic vasculitis may be punctuated by acute and life-threatening manifestations that require management in an intensive care unit (ICU). The diagnosis may be delayed or completely unrecognized. The six cases reported here illustrate the challenges raised by the diagnosis and management of a specific type of small-vessels vasculitis, i.e.,cryoglobulinemic vasculitis (CV), particularly in the ICU. CV usually runs a slow course, but acute flares with life-threatening manifestations may occur. A high level of suspicion is essential, and all CV patients should be monitored closely to ensure a timely and accurate diagnosis and to initiate appropriate treatment. Acute respiratory distress and AKI were the main reasons for ICU admission in our patients. Other serious manifestations that should be considered are involvement of the lungs, heart, central nervous system, and gut. Patients usually present with multiorgan failure, and the possibility of widespread vasculitis should be considered. Identification of the underlying cause is crucial, because adding etiology-based treatment to the life-supporting interventions and immunosuppressive therapy is likely to improve the outcome.

## Abbreviations

AKI: Acute kidney injury; CT: Computed tomography; CV: Cryoglobulinemic vasculitis; HCV: Hepatitis C virus; Ig(s): Immunoglobulin(s); ICU: Intensive care unit.

## Competing interests

The authors declare that they have no competing interests.

## Author’s contributions

MZ, EM, LG, BA, AM, and EA participated in the recruitment of the patients used to illustrate the review and in the design of the manuscript. VM and JV provided the illustrations and performed the pathological analysis of biopsy specimens. MZ, EM, AM, and EA drafted the initial manuscript. All authors read and approved the final manuscript.

## Supplementary Material

Additional file 1: Table S1 Classification of the cryoglobulins (adapted from Ferri C. Mixed cryoglobulinemia. Orphanet J Rare Dis 2008; 3:25) [1,4]. **Table S2.** Main clinical features of Type II mixed cryoglobulinemia [1-6]. Click here for file
