# Supplementary material for: Vasculitic emergencies in the intensive care unit: a special focus on cryoglobulinemic vasculitis
Source: Ann Intensive Care. 2012 Jul 19;2:31. doi: 10.1186/2110-5820-2-31 (PMC3488028; doi:10.1186/2110-5820-2-31)
Supplement: Additional file 1: Table S1 — Classification of the cryoglobulins (adapted from Ferri C. Mixed cryoglobulinemia. Orphanet J Rare Dis 2008; 3:25) [1,4]. Table S2. Main clinical features of Type II mixed cryoglobulinemia [1-6]. [file 2110-5820-2-31-S1.doc]

**Supplementary Table 1. Classification of the cryoglobulins (adapted from Ferri C. Mixed cryoglobulinemia. Orphanet J Rare Dis 2008; 3:25) [1, 4]**.

| **Cryoglobulinemia** | **Composition** | **Underlying conditions** |
| --- | --- | --- |
| **Type I**  10-15% | monoclonal Ig (mainly IgG or IgM) | -lymphoproliferative disorders (CLL, B-cell NHL, MM, WM). |
| **Type II (mixed)**  50-60% | monoclonal Ig (typically IgM *kappa*) with RF activity and polyclonal IgGs | -infections (mainly HCV)  -autoimmune disorders  -lymphoproliferative disorders (type II > III )  - essential (no identifiable cause) |
| **Type III (mixed)**  30-40% | polyclonal mixed Ig with RF activity of one polyclonal component (usually IgM) |
| MM, multiple myeloma; WM, Waldenström’s macroglobulinemia; CLL, chronic lymphocytic leukemia; NHL, non-Hodgkin's lymphoma; Ig, immunoglobulin; RF, rheumatoid factor; HCV, hepatitis C virus | | |

**Supplementary Table 2.** **Main clinical features of Type II mixed cryoglobulinemia [1-6].**

| **MAIN CLINICAL FEATURES OF MIXED CRYOGLOBULINEMIA** | |
| --- | --- |
| **General signs** | Weakness (70-98%)  Fever (5%) |
| **Skin lesions** | Purpura (>50-90%)  Ulcers (10%)  Raynaud phenomenon ± digital gangrene (30%)  Other (skin rash, livedo reticularis, acrocyanosis) (5%) |
| **Joint manifestations** | Arthralgia and myalgia (90%)  Nonerosive arthritis (8%) |
| **Peripheral neuropathy** | Sensory or mixed polyneuritis (60-80%)  sensory-motor (asymmetric) mononeuritis (rare) |
| **Sicca syndrome** | Xerostomia, xerophtalmia (30-50%) |
| **Renal involvement** | Proteinuria, acute kidney injury, progressive renal failure (20-30%)  Glomerulonephritis (invariably MPGN) |
| **B-cell lymphoproliferative disorders** | Non-Hodgkin Lymphomas (5-10%): according to the REAL/WHO classification, the most frequent histologic subtypes are  - lymphoplasmacytic  - diffuse large B-cell  - follicular  - marginal zone  - mantle cell lymphomas |
| **HCV-related liver involvement** | Chronic hepatitis, Cirrhosis (25-50%)  Hepatocellular carcinoma (3%) |
| **MAIN BIOLOGICAL FEATURES OF MIXED CRYOGLOBULINEMIA** | |
| **Type II/III mixed cryoglobulins** | 2/1 |
| **Low C3** | 20% |
| **Low C4** | 65% |
| **Rheumatoid factor** | 95-100% |
| **Antinuclear antibodies** | 30% |
| **Anti-ENA antibodies** | 8% |
| **anti-HCV antibodies ± HCV RNA** | 92% |
| **Anti-HBV antibodies** | 32% |
| HCV, hepatitis C virus; MPGN, membranoproliferative glomerulonephritis; ENA, extractable nuclear antigen; HBV, hepatitis B virus. | |
